# Shedding Light on Perioperative Nerve Injury: An Investigation Using the SHED (Symptoms Categorization-History Taking-Examination-Diagnostic Evaluations) Approach

**DOI:** 10.7759/cureus.54133

**Published:** 2024-02-13

**Authors:** Kartik Sonawane, Pratiksha Rao, Haripriya T., Tuhin Mistry, Chelliah Sekar

**Affiliations:** 1 Anesthesiology, Ganga Medical Centre and Hospitals, Pvt. Ltd., Coimbatore, IND

**Keywords:** pain management, regional anesthesia, peripheral nerve block, neuropraxia, nerve injuries, peripheral nerve injuries

## Abstract

Peripheral nerve blocks (PNBs) provide analgesia and anesthesia in diverse surgical procedures. Despite their recognized benefits, the occurrence of complications, particularly peripheral nerve injuries (PNIs), is a noteworthy concern. Prompt identification and intervention for perioperative nerve injuries are crucial to prevent permanent neurological impairment. A meticulous, systematic evaluation centered on the onset and progression of symptoms becomes imperative. The SHED (symptoms categorization-history taking-examination-diagnostic evaluations) approach serves as a valuable tool for diagnosing causative factors, determining the type of nerve injury, and formulating an effective treatment plan to mitigate further harm.

This case report employs the SHED approach to elucidate a perplexing instance of PNIs. The patient, experiencing neurological symptoms post-forearm surgery under a PNB, serves as a focal point. The report underscores the significance of a systematic, stepwise approach in managing patients with suspected PNIs. Vigilant patient monitoring, collaborative teamwork, shared responsibilities, and consideration of potential contributing factors beyond the nerve block are highlighted for an accurate diagnosis and effective treatment of PNIs. The aim is to guide healthcare professionals in navigating similar clinical scenarios, ultimately ensuring patient safety and optimizing outcomes.

## Introduction

Peripheral nerve blocks (PNBs) have emerged as a fundamental component of contemporary anesthesia practices, offering effective analgesia and anesthesia for diverse surgical interventions. Their utilization is associated with notable benefits, including diminished opioid consumption, enhanced postoperative pain control, and potential reductions in hospital stays. However, as with any medical procedure, PNBs carry inherent risks. One infrequent yet significant complication is peripheral nerve injury (PNI), which may lead to enduring sensory or motor deficits, significantly impacting a patient’s overall quality of life.

In the perioperative period, PNI occurs rarely, with reported incidences ranging from 0.03% to 0.1% [1]. Unraveling its multifactorial pathogenesis proves challenging [2], with factors such as hypovolemia, dehydration, hypotension, hypoxia, electrolyte imbalances, and hypothermia contributing to perioperative PNIs [3]. Newly developed sensory or motor deficits or paresthesia without apparent causes may manifest within five days postoperatively, persisting for over 10 days [4,5]. These occurrences can be linked to patient comorbidities, positioning during surgery, and specific surgical conditions.

Despite PNIs being most commonly associated with surgical causes [6,7], they rank as the primary and third most frequent causes of anesthesia-related litigation [8]. Consequently, perioperative neurological deficits pose a significant challenge, necessitating a comprehensive, step-by-step assessment. The sudden onset of unexpected symptoms prompts numerous questions: what is the cause? Could it be related to PNBs? Are there alternative explanations? What steps should be taken next? How can the condition be evaluated and addressed? Will spontaneous recovery occur, or does it warrant specific interventions and consultations? Addressing these inquiries is not only vital for medical considerations but also holds legal implications.

While existing literature outlines nerve injuries, categorizes them, and provides guidance on necessary consultations, examinations, or medications, ambiguity persists regarding precise courses of action. Effectively managing such cases requires a systematic approach to determine causative factors, select appropriate diagnostic evaluations, and diagnose the type of PNI based on symptomatology. This case report emphasizes the significance of the SHED (symptoms categorization-history taking-examination-diagnostic evaluations) approach [9] as a stepwise method for diagnosing and managing patients with suspected PNIs. Having provided written informed consent for surgical procedures under regional anesthesia (RA), the patient agreed to disclose case-specific details while maintaining confidentiality and allowing the publication of this case report.

## Case presentation

**Figure 1:**
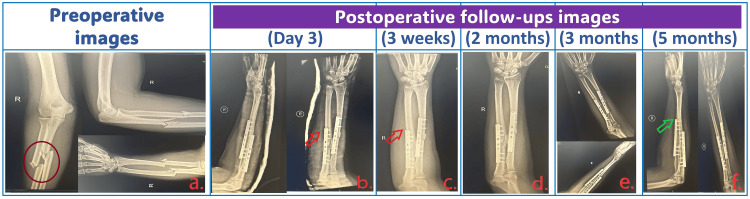
Preoperative and postoperative radiographic follow-up images. a. Preoperative radiographic images showing comminuted fractures of both right forearm bones. b. Postoperative radiographic images (on POD3) showing increased soft tissue swelling around the operated site. c. Postoperative follow-up radiographic images at three weeks. d. Postoperative follow-up radiographic image at two months. e. Postoperative follow-up radiographic image at three months. Images c-e show decreased soft tissue swelling around the operated site. f. Postoperative follow-up radiographic images (at five months) showing normal soft tissues without swelling around the operated site. POD: postoperative day; dark brown circle in (a): involved fractured site of the right forearm; red arrow: soft tissue swelling around the operated site; green arrow: showing normal soft tissues (without swelling) around the operated site

A 32-year-old healthy male driver was admitted to the emergency room with a closed comminuted fracture of both bones of the right forearm (Figure 1, Panel a), attributed to a fall from a two-wheeler. He was scheduled for an open reduction and internal fixation of both bones with screws and locking plates under ultrasound-guided right costoclavicular block (CCB) and autologous iliac crest bone grafting under spinal anesthesia (SA). After discussing the benefits and risks, the patient provided consent for surgery under RA (CCB + SA) and an additional right quadratus lumborum block (QLB) for postoperative analgesia of bone grafting.

Preoperatively, the patient received premedication consisting of intravenous midazolam 2 mg, pantoprazole 40 mg, and ramosetron 0.3 mg. Ultrasound-guided right CCB was administered using 20 mL of a local anesthetic (LA) mixture (0.75% ropivacaine + 8 mg dexamethasone). Before the CCB, the patient had severe pain with a score of 7/10 on a numeric rating scale (NRS) in the right forearm. Twenty minutes after CCB, his pain scores decreased to 0/10 on NRS. Thirty minutes after CCB, the patient was shifted to the operating room (OR) upon verifying the complete loss of pinprick and cold sensations at the intended surgical site.

Intraoperatively, a subarachnoid block was administered (for bone graft retrieval) at the L3-L4 space using 0.5% hyperbaric bupivacaine with the patient kept in a sitting position and connected to the standard American Society of Anesthesiologists (ASA) monitors. The patient was then placed in the supine position for surgery with the tourniquet applied to the right arm. Throughout the surgical procedure, maintaining the patient’s comfort and well-being was of paramount importance. Regular communication was upheld, and any concerns or complaints expressed by the patient were promptly addressed. Multimodal analgesia (MMA) was instituted in the form of intravenous paracetamol 1 g, ketorolac 30 mg, and dexamethasone 8 mg. The patient remained comfortable, pain-free, and awake throughout the procedure of about 3.5 hours, with a blood loss of around 200 mL. Immediately following surgery, ultrasound-guided right QLB was administered using 30 mL of LA mixture (0.2% ropivacaine + 8 mg dexamethasone) to provide postoperative analgesia over the bone graft retrieval site.

**Figure 2:**
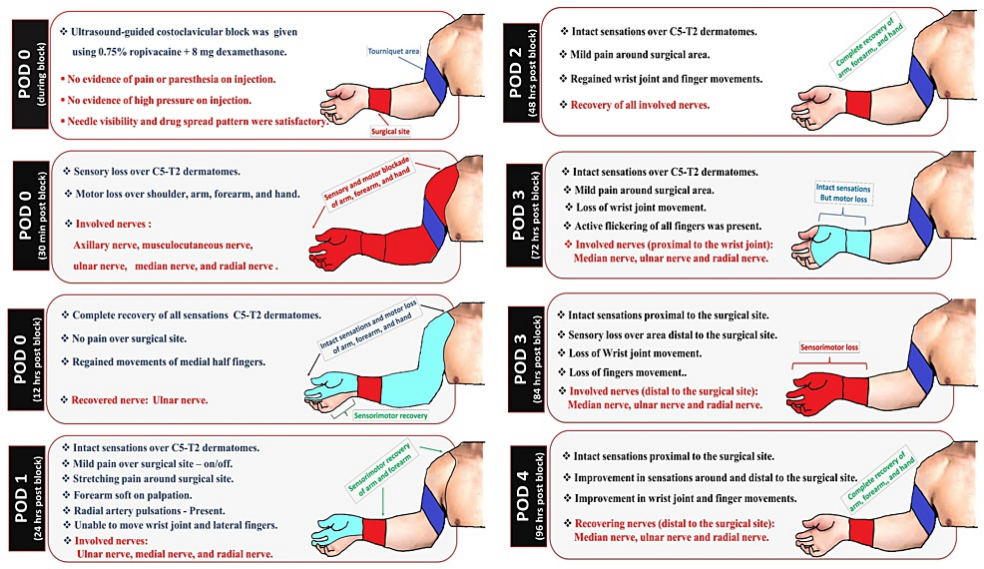
Evolution and recovery dynamics of perioperative neurological deficits. This figure was created by the first author (KS). POD: postoperative day

Postoperatively, the patient was monitored in the recovery room for two hours. He was encouraged to resume oral intake within an hour following surgery. Postoperative MMA protocol included oral paracetamol 1 g four times daily, aceclofenac 100 mg twice daily, and pregabalin 75 mg at bedtime. As a routine protocol, the patient was monitored regularly to assess recovery (Figure 2) from the administered RA techniques. The patient recovered completely from the neuraxial block six hours after surgery. Twelve hours post-CCB, he showed sensory recovery of all dermatomal regions (C5-T2) of the right upper extremity with partial motor recovery of the medial fingers (ulnar nerve). There was occasional pain (2/10 on NRS) when stretching at the surgical site. He recovered completely from the sensorimotor effect of the CCB on the second postoperative day (POD).

On POD 3, he developed motor weakness in the wrist joint with preserved finger movements that progressed to complete sensorimotor loss distal to the surgical site after a few hours. After removing the cast, the surgical wound was assessed for swelling, discoloration, or bleeding. Assessment of the block site revealed no tenderness, swelling, or discoloration. His symptoms subsequently improved, and the affected nerves recovered the next day (POD 4). Upon reassurance of neurological recovery and the absence of adverse effects, the patient was discharged on POD 5 with a pain score of 2/10 on the NRS.

Management

Upon observing unexpected neurological symptoms on POD 3, a prompt neurological consultation was sought to comprehensively assess the patient’s condition. Discussions with the surgical team ensued, contemplating the necessity of surgical reexploration to investigate potential hematoma, nerve impingement, or vascular compromise. Considering the evolving recovery pattern of the symptoms, nerve conduction studies (NCS) and electromyography (EMG) were judiciously deferred. Instead, a pragmatic approach was adopted, and the decision was made to enhance the ongoing MMA by incorporating intravenous dexamethasone 8 mg thrice daily. This addition aimed to mitigate inflammation and optimize the patient’s neurological recovery.

In collaboration with physical therapy and other conservative measures, including intravenous steroids, the patient’s deficits exhibited significant improvement over the subsequent 72 hours. Vigilant monitoring, reassurance, and a holistic approach to care played pivotal roles in achieving positive outcomes. The decision to refrain from surgical reexploration proved sound as the patient responded favorably to the non-invasive interventions, underscoring the importance of a multidisciplinary approach in managing unexpected neurological sequelae. Subsequent clinical and radiological follow-ups (Figure 1, Panels b-e) showed complete resolution of neurological symptoms and gradual reduction in pain and soft tissue edema.

## Discussion

**Figure 3:**
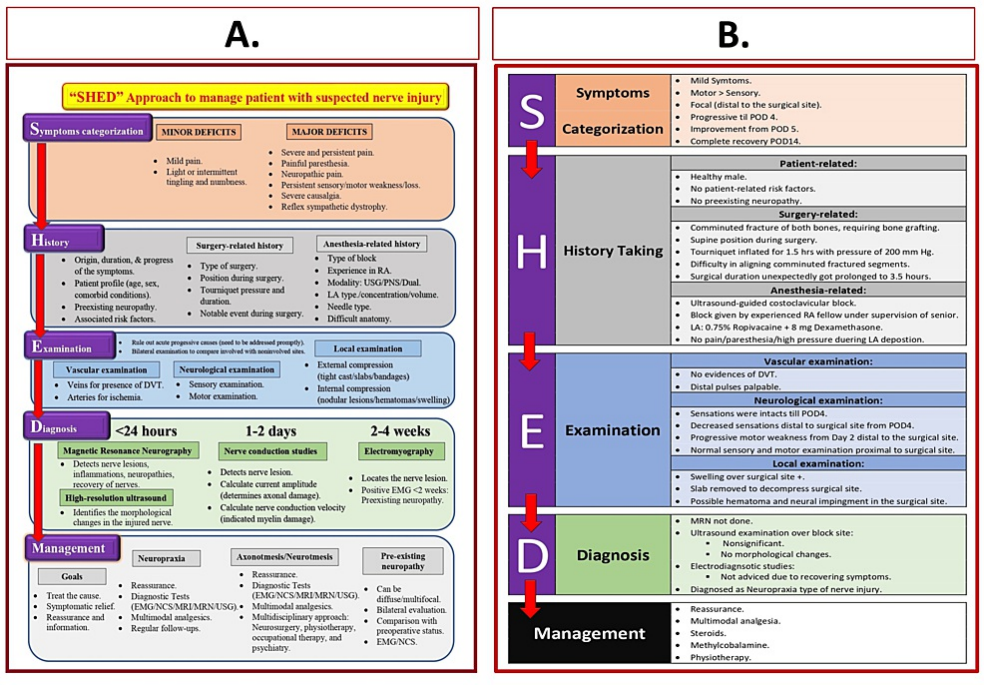
SHED approach to manage perioperative nerve injury. A: Steps of the SHED approach [9]. B: Incorporation of the SHED approach to managing patients with perioperative nerve injury. Source: This figure was created by the first author (KS). RA: regional anesthesia; POD: postoperative day; LA: local anesthetic; USG: ultrasound guidance; PNS: peripheral nerve stimulator; DVT: deep venous thrombosis; EMG: electromyography; NCS: nerve conduction studies; MRI: magnetic resonance imaging; MRN: magnetic resonance neurography

Nerve injuries following PNBs represent a recognized albeit rare complication. In our patient, the precise mechanism leading to neurodeficit was initially elusive, emphasizing the need for a systematic approach. Adopting the SHED approach (Figure 3), we aimed to identify causative factors and devise a comprehensive management plan.

The SHED approach facilitated the exploration of potential causes, categorizing them into block-related factors (such as direct needle trauma, intraneural injection, or local anesthetic toxicity), pressure-related factors (including compression of the nerve by a tourniquet, surgical-site hematoma, or postsurgical edema), and surgery-related factors (encompassing direct nerve injury during surgery or nerve impingement during retraction or implant placement) [10]. By utilizing this framework, we pursued a methodical examination of each potential contributor.

Our strategy involved a meticulous exclusion method, systematically ruling out each potential cause to unveil the underlying factors contributing to the neurodeficits. This approach not only guided our diagnostic efforts but also paved the way for a targeted and informed management plan. Incorporating the SHED approach played a pivotal role in navigating the complexity of nerve injuries post-PNBs, allowing for a precise understanding of causative elements and shaping the subsequent course of action.

Symptom categorization

Sensory manifestations following nerve injury encompass a spectrum, including anesthesia, paresthesia, hypoesthesia, hyperesthesia, and pain within the affected nerve territory [3]. Paresthesia, indicative of heightened spontaneous nerve activity following an initial insult, often manifests in the aftermath of nerve injury [11]. Motor symptoms involve muscle weakness, ranging from mild paresis to complete paralysis. Systematic assessment of sensorimotor deficits aids in determining the type and severity of nerve or plexus injury. Our patient had a rapid and complete recovery of sensory function within 12 hours and motor function within 48 hours post-block. Subsequently, the emergence of focal symptoms, primarily motor, localized distally to the surgical site, displayed an on/off pattern. Notably, symptoms demonstrated improvement from POD 4, culminating in complete resolution by POD 14. The transient nature of the symptoms suggested a mild deficit category, indicative of a favorable prognosis with anticipated resolution within days or weeks, obviating the need for extensive diagnostic evaluations. On the other hand, major deficits are persistent and severe, warranting the need for additional diagnostic investigations, consultations, and ongoing supportive care.

History taking

A detailed medical history revealed the patient’s overall health without comorbidities or preexisting neurological symptoms. Comorbid conditions play a crucial role in perioperative PNIs [1]. The administration of ultrasound-guided CCB with 0.75% ropivacaine was uneventful, with no documented evidence of neural damage during injection. Using real-time ultrasound guidance in RA contributes to enhanced safety [12], minimizing the risk of adverse events. It has been shown to have several advantages in nerve localization, including speed of block onset and block success rate [13]. Possible mechanisms of nerve injuries during ultrasound guidance include mechanical trauma, ischemia, or chemical neurotoxicity [6]. All LAs possess concentration-dependent neurotoxic potential and can cause focal demyelination and axonal destruction [14,15]. However, the absence of block-related complications during RA administration ruled out direct needle trauma, intraneural injection, or LA toxicity as causes of the observed nerve injury. The possibility of nerve injury attributed to the block technique, LA, or the needle seemed unlikely, considering the patient’s swift recovery of all sensations and movements distal to the block site. However, the incidence of PNIs following plexus block is estimated to be 0.01% to 14% [7,16-18].

Further, the rarity of tourniquet-related PNIs was considered, with an estimated overall incidence of 0.01-0.02% [19]. Safe tourniquet duration and pressure parameters were maintained during surgery, minimizing the likelihood of tourniquet-related PNIs. The patient’s supine position during surgery eliminated position-related injury risks. However, the intricate nature of the fracture led to challenges during surgical alignment, resulting in extensive soft tissue manipulation and prolonged surgical duration. History taking pointed toward potential surgery-related causes, necessitating focused clinical examination to confirm suspicions. Surgery-related mechanisms causing PNIs include crushing, stretching, contusion, laceration, transection, or incorporation of a nerve in the suture repair. During hand or forearm surgery, nerves (musculocutaneous, median, ulnar, and radial) can get injured due to traction, crushing, and lacerations [20].

Examination

Vascular examination revealed no evidence of skin color changes or absence of pulsations distal to the surgical site. Local examination of the involved sites (PNB, frequent entrapment, tourniquet, and surgery) was pivotal in ruling out external/internal compression factors on neural/vascular structures. The patient’s neurodeficit limited to the area distal to the surgical site suggested potential surgical causes of nerve injury, ruling out position-related and tourniquet-related factors.

Therefore, our focus shifted to exploring possible surgery-related causes such as nerve impingement or compression due to hematoma or edema formation or direct nerve injury during surgical dissection. Restoration of all sensations on POD 2 negated the risk of direct surgical nerve injury. The subsequent involvement of the area distal to the surgical site suggested the possibility of compressive neuropathy due to hematoma or edema formation or possibly postsurgical inflammatory neuropathy (PSIN). Neuropathy due to compressive hematomas, unlike spinal or epidural hematoma, typically resolves completely. However, it is common in patients on anticoagulants [21]. The presence of a surgical drain and the absence of anticoagulants in our patient excluded the possibility of compressive hematoma. Our patient’s neuropathy close to the surgical site (not remote) with immediate onset (not delayed) excluded the possibility of immune-mediated neural tissue inflammation (PSIN) [22]. Ultimately, edema formation due to extensive soft tissue handling might be the cause of compressive neuropathy in our patient.

Diagnosis

The pattern of onset, progression, and improvement of neurological symptoms in our patient suggested neuropraxia-type nerve injury, mainly due to surgical causes. Reversible conduction blockade in neurapraxia is due to localized myelin sheath degeneration or segmented demyelination. Most neurological injuries related to PNBs (greater than 90%) generally resolve within four to six weeks, and over 99% resolve by one year [17,23]. Perioperative neurapraxia is mainly an entrapment neuropathy resulting from transient or intermittent ischemia that improves quicker than focal demyelination. Axonotmesis is often caused by stretching or crushing injuries and may take months or years to recover fully or partially [9,10]. Therefore, nerve injuries such as axonotmesis or neurotmesis confer real morbidity and further diagnostic and therapeutic interventions [9,10]. In contrast, neuropraxia recovers completely within days to weeks (12 weeks) [9,10] without further diagnostic evaluation. As our patient exhibited complete symptom improvement within seven days (POD 4), further diagnostic studies, such as electrodiagnostic studies (NCS and EMG), were deemed unnecessary.

Electrodiagnostic studies (NCS and EMG) help diagnose nerve injury types and their prognostication. NCS detects nerve lesions, and the EMG study locates them. Motor and sensory axons remain excitable for 7-11 days [9], and Wallerian degeneration begins after 10-12 days [24]. Therefore, an initial (1-2 days) and follow-up NCS (10-14 days) after nerve injury are recommended to decide upon the types of nerve injuries. Abnormal spontaneous electrical activity within the affected muscle takes one to four weeks to develop and disappears with reinnervation [9,10]. Therefore, an initial EMG study is recommended within two to four weeks following nerve injuries [25]. However, our patient recovered beforehand, eliminating the need for such diagnostic studies.

Management

The SHED approach suggests a wait-and-watch strategy for neuropraxic injuries with continued conservative treatment and scheduled follow-up assessments to monitor symptom progression or recovery. Due to persistent sensorimotor complaints, we sought a neurological consultation to ensure a comprehensive evaluation. Neurological consultations are typically reserved for unresolved neuropraxia beyond one month, persistent sensory deficits lasting over five days, or when the motor component is involved [9]. Simultaneous engagement with physical and occupational therapy consultations was crucial for preventing contractures, muscular atrophy, and ongoing disability. The patient exhibited complete symptom resolution within 14 days post-nerve injury, underscoring the effectiveness of the multimodal approach to analgesia, steroids, and physiotherapy.

Indeed, the management of perioperative nerve injuries involves a multifaceted approach, covering conservative methods, diagnostic and therapeutic interventions, and supplementary strategies such as physiotherapy, rehabilitation, and psychological support. What sets this case report apart is its emphasis on the significance of a systematic, stepwise approach for both diagnosis and concurrent management of perioperative nerve injuries. Furthermore, it underscores the importance of assigning responsibilities clearly within the healthcare team. This approach can significantly enhance the care and outcomes for patients experiencing such injuries.

## Conclusions

The SHED approach offers a comprehensive framework wherein potential nerve injuries can be thoroughly examined, categorized, and concurrently diagnosed and treated. Each step within this approach contributes to accumulating positive findings, necessitating confirmation in subsequent stages before definitive management protocols can be established based on the final diagnosis. This method advocates vigilant monitoring, reassurance, and conservative treatment for symptoms in recovery, thereby conserving resources and circumventing unnecessary interventions. In contrast, escalating symptoms prompt immediate neurosurgical consultations and electrodiagnostic studies to prognosticate nerve injuries. The potential for nerve injury associated with PNBs should not impede the refinement of RA practices or the adoption of safe patient-centric, tailor-made, and procedure-specific RA techniques.
